# Who is the emergency care community? A qualitative exploration of stakeholder roles and relationships in engaged research within a South African provincial emergency care system

**DOI:** 10.1016/j.afjem.2026.100950

**Published:** 2026-02-04

**Authors:** Robert Holliman, Willem Stassen, Colleen Saunders

**Affiliations:** Division of Emergency Medicine, University of Cape Town

**Keywords:** Community, Engaged research, Engaged scholarship, Frontline clinicians

## Abstract

**Introduction:**

Effective emergency care (EC) in low- and middle-income countries (LMICs) depends on evidence that is relevant, implementable, and locally informed. Engaged research offers a means to achieve this, yet practical understanding of how to identify and involve communities remains limited. Defining the “community” is a critical but underexplored step, and little is known about how engagement operates within LMIC EC systems. This study explored stakeholder perspectives on the composition and relationships of the EC community.

**Method:**

This was a qualitative study using semi-structured interviews and descriptive content analysis exploring stakeholder perspectives on the EC community. Purposive and snowball sampling ensured diverse representation. Transcripts were analysed inductively drawing on Braun and Clarke’s thematic framework and reported in accordance with COREQ guidelines.

**Results:**

Thirty stakeholders participated, including emergency physicians, nurses, prehospital providers, academics, policymakers, and community representatives, many holding overlapping roles. Analysis identified three overarching categories:(1) breadth, variability, and ambiguity within the community;(2) fragmentation and exclusion; and (3) engagement potential and the role of frontline clinicians.

**Conclusions:**

This study highlights the complexity of defining the EC community, with boundaries that are broad, variable, and often contested. Participants identified the absence or unclear roles of key stakeholders, including patients, decision-makers, and frontline clinicians, within research processes. Fragmentation and limited trust were viewed as major barriers to meaningful engagement, reinforcing the need for stronger, more transparent relationships within the community. Frontline clinicians were consistently recognised as underutilised yet uniquely positioned to connect clinical realities with academic research and community priorities. Strengthening engaged research in this context will require clearer guidance on how and when to involve stakeholders, structured mechanisms to build and sustain trust, and practical support for clinician participation. Together, these steps could help embed engagement more deeply into research culture and enhance the relevance and impact of EC scholarship.

## African Relevance

• Engaged research is vital for improving knowledge translation and production in African emergency care
• Mapping emergency care stakeholders is key to meaningful engagement in African settings
  Research must reflect real priorities given limited research capacity in Africa
• Understanding emergency care actors supports more relevant and inclusive scholarship

## Introduction

The delivery of effective, high-quality emergency care (EC) depends on practice, guidelines, and systems informed by the best available evidence [1]. This is particularly important in low- and middle-income countries (LMICs), where there is a well-documented mismatch between disease burden and available resources [2]. However, for evidence to be meaningful, it must be both implementable and transferable to local settings and populations. This presents a challenge for EC providers in LMICs, as most available evidence is derived from research conducted in high-income countries (HICs), often focused on advanced technologies and disease profiles unrepresentative of LMIC populations.

Poor translation of research findings into routine clinical practice, along with significant delays in the adoption of evidence, continues to hamper progress in healthcare delivery [3]. These challenges are further compounded for LMIC EC professionals, who face well-documented, multifactorial barriers to undertaking research (e.g. limited funding, expertise and protected time) and to participating in the wider research ecosystem (e.g. costs of conference attendance, visa and travel barriers, and the lower visibility of LMIC outputs) [[4], [5], [6], [7]]. For these reasons, the research that is conducted in these settings must be focused and aligned to local needs, to generate knowledge that meaningfully benefits the communities it aims to serve.

In response to these challenges, there has been a growing shift toward more collaborative forms of scholarship that engage non-academic stakeholders not only in conducting research, but throughout the entire research process, including topic selection, study design, and dissemination of findings [8]. These approaches, often described under the umbrella of *engaged scholarship*, have been associated with improved translation of findings into clinical practice [[8], [9], [10]], as well as broader benefits such as leadership development, workforce strengthening, and enhanced institutional capacity [11]. Furthermore, there is evidence that decision-makers are more likely to support and adopt research when they have been involved in the project development or funding [12].

To engage meaningfully with a specific community, there must first be an understanding of who its key stakeholders are. The concept of “community” is itself contentious, with extensive research devoted to unpacking this ambiguous term [[13], [14], [15]]. Broadly, a community can be understood as a group of people connected by a shared perspective who engage in joint action, often within a defined geographic setting [14]. To support stakeholder-centred research within such communities, frameworks like the *Seven Ps* stakeholder taxonomy (*Patients and the Public, Providers, Purchasers, Payers, Policy Makers, Product Makers, and Principal Investigators*) have been developed [16]. These frameworks are specifically designed to be adaptable and serve as prompts to help identify relevant stakeholders in a given context. However, it remains essential to recognise that each community is shaped by unique relationships and local dynamics. As such, stakeholder identification must still be grounded in an understanding of the specific setting, with careful attention to defining the community’s boundaries and composition before engagement efforts are undertaken.

Although the speciality of emergency medicine (EM) is well established in HICs, it remains relatively new in many LMICs, particularly in Africa. As of 2019, only 15 formal EM physician training programmes had been recognised across 12 African countries [17], with the earliest established in the Western Cape (WC), South Africa, in 2004 [18]. This limitation extends beyond physicians to other EC providers. Nurses form the backbone of frontline EC and are responsible for a large proportion of patient assessment and ongoing management [19], yet formal emergency nursing education and role development remain uneven across the continent [20,21]. Similarly, prehospital care systems in many LMICs rely on personnel with limited access to structured EC training, even though prehospital providers are critical first responders and key interfaces between communities and health facilities [21].

Despite South Africa’s upper middle-income World Bank classification, high levels of poverty exist and while the WC has comparatively higher living standards than most LMICs, it remains economically polarised, and substantial socio-economic challenges place continued strain on its public EC services [22]. The WC EC research community is among the most productive in Africa [23] situated within a local context in which engaged scholarship has been increasingly recognised and actively developed [24]. In South Africa, engagement is increasingly valued not only for enhancing research relevance and impact, but also as a means of addressing structural and socio-economic challenges. Although national policies have recognised its importance since the 1990s, including through key national White Papers [8], implementation remains uneven across disciplines and institutions.

Within EC, efforts to strengthen engaged scholarship are often limited by the absence of a clearly defined community and stakeholders. Advancing meaningful engagement therefore requires a clearer understanding of who constitutes the EC community, who the key stakeholders are, and how they relate to one another. This study aimed to explore perceptions of the WC EC community, including its key actors, boundaries, and stakeholder relationships, to inform future engagement efforts.

## Methods

### Study design

This study was positioned within a relativist, constructivist paradigm, based on the philosophical assumption that reality is socially constructed. It utilised semi-structured interviews (SSIs) and descriptive content analysis to explore stakeholder perspectives on who constitutes the community, the current state of engagement between its members, and their experiences with engaged scholarship. Initial interviews informed iterative changes to the SSI guide, ensuring subsequent interviews reflected emerging categories. This study is reported in accordance with the Consolidated Criteria for Reporting Qualitative Research (COREQ) checklist [25]. Ethical approval was obtained from the Human Research Ethics Committee of the University of Cape Town (HREC REF: 403/2023).

### Setting

The WC is one of nine provinces in South Africa and is home to approximately 7.1 million people, making it the third most populous province [26]. The WC reflects South Africa’s broader health profile, which is characterised by a quadruple burden of disease: HIV/AIDS and other sexually transmitted infections; non-communicable diseases, including mental health conditions; maternal, neonatal and child health concerns; and tuberculosis, alongside a significant burden of interpersonal violence and injuries [27]. EC in the province is delivered through both public and private sectors, with roughly three-quarters of the population reliant on public health services [27]. The public health system includes a wide network of primary care facilities, hospitals, and emergency medical services. While primary healthcare serves as the main point of entry, EC is delivered across the system by emergency physicians, nurses, and prehospital providers.

### Study population and sampling strategy

Following approval from relevant institutions and organisations, identified individuals were contacted via email and invited to participate in SSIs. To ensure a broad and inclusive range of perspectives from the WC EC community, we adopted a two-stage sampling strategy. In the first stage, we employed purposive sampling to identify and invite key individuals representing the full spectrum of stakeholder groups within the EC system, guided by the *Seven Ps framework* [16]. Following the initial round of interviews, we implemented snowball sampling to identify additional participants [28]. Interviewees were asked to suggest other individuals or groups who they believed held relevant insights or played important roles in the EC community, particularly those who may not be readily visible or accessible through formal networks.

### Author characteristics and credentials

*RH* is a UK-trained EM specialist who has worked within the WC EC community since 2020. He is currently undertaking a PhD in EM and holds an MBBS and BSc. *WS* is an Associate Professor, holds a PhD in EM, and is a convenes a PhD in EM programme in the WC. *WS* and *RH* identify as men and have clinical backgrounds in EM and paramedicine, respectively. *CS* is an Associate Professor in the WC, where she leads the Departmental research portfolio. She holds a PhD and is female. *WS* is an experienced qualitative researcher who has previously led several qualitative projects and authored multiple publications. *RH* and *CS* had developing experience in qualitative research at the outset of this study, with their involvement closely guided and supported by *WS* throughout this project.

### Data collection

Data were collected through SSIs conducted via an online platform (Microsoft Teams, Microsoft Corporation, Redmond, Washington, U.S.) and audio recorded. Interviews were conducted by the primary researcher (*RH*), with *CS* or *WS* attending initial interviews to observe and guide early discussions. Interviews followed a discussion schedule developed from a review of literature on engaged scholarship and adjusted iteratively throughout the process based on participant feedback and emerging insights (appendix A). Initial interviews were conducted with *CS* and *WS* to serve as pilots, with the answers included in the data analysis and used to refine the discussion schedule.

Interviews explored participants’ understanding and experiences of engaged scholarship within the WC, perceptions of who constitutes the community in the WC EC context, and views on current relationships within this community. All interviews were conducted in English and transcribed verbatim by *RH*. Field notes were taken during interviews to support the analysis.

### Data analysis

Transcripts were analysed using inductive-dominant content analysis to the manifest level, supported by NVivo Pro (v.14; QSR International, Burlington, Massachusetts, U.S.). The analytic approach drew on the initial six phases of Braun and Clarke’s thematic analysis framework: familiarisation with the data, initial coding, initial categorising, category review, category defining and naming, and reporting [29].

Code development was an iterative process, allowing for continuous refinement as new patterns emerged. Following analysis of the initial interviews, a preliminary code set was developed. Subsequent transcripts were analysed using a combined inductive–deductive approach, with new codes created inductively as needed and existing codes applied deductively across transcripts to ensure consistency. Once coding was complete, related codes were clustered into higher-order conceptual groupings based on shared meaning. These groupings were then examined and refined through iterative discussion among all authors to ensure internal coherence and clear distinction, resulting in the final analytic categories used to structure the findings. Initial coding was conducted by *RH*, with codes reviewed and refined with *WS* and *CS*, and category development undertaken collaboratively by the research team.

### Trustworthiness

The Guba framework of credibility, transferability, dependability, and confirmability was used to guide and enhance qualitative rigour [30]. *Credibility* was supported by drawing on established methods and prior literature to inform the interview structure, coding categories, and analytic approach. Informed consent procedures ensured participants understood the study’s purpose, and one-on-one interviews promoted openness and honesty. Iterative questioning was employed during interviews, and post-interview debriefings among the research team (*RH, WS & CS*) provided a space to reflect on emerging ideas and assumptions, further contributing to credibility [31]. *Transferability* was addressed by clearly describing the study setting, enabling readers to consider the relevance and applicability of findings to their own contexts. *Dependability* was strengthened through transparent documentation of study procedures, including detailed reporting of data collection and analysis methods, supporting future replication or adaptation [30,31]. *Confirmability* was enhanced by regular team debriefings and ongoing reflective commentary. A detailed description of the SSI and analysis process is provided, and the COREQ checklist was used to ensure transparency and support both confirmability and dependability [25].

## Results

Nineteen stakeholders initially agreed to participate in the study. Following these interviews, an additional eleven individuals were identified through snowball sampling, recruited, and interviewed, resulting in a total of 30 interviews. No participants who were directly approached declined to participate, although a small number (estimated three to five) did not respond to invitations. No participants withdrew after consenting to be interviewed. Eighteen participants self-identified as women and 12 as men. Interviews lasted between 36 and 66 minutes, with an average duration of 51 minutes. No repeat interviews were carried out. Many participants held joint or multiple roles across clinical, academic, and managerial domains, making it difficult to provide a precise count for each professional category. The cohort did, however, include prehospital providers, EM physicians and nurses, academics, managers and policy-makers, patient advocacy group leaders, patient community representatives, and postgraduate degree researchers. Participant demographic information is summarised in Table 1.

**Table 1:** Participant demographic info.

Participant No.	Gender	Clinical Background	Current Role	Sector
P1	Male	Prehospital provider	Researcher	Public
P2	Female	None	Researcher	Public
P3	Male	Emergency Physician	Joint staff: Clinical & Academic	Public
P4	Female	Emergency Physician	Joint staff: Clinical & Academic	Public
P5	Male	Emergency Physician	Joint staff: Clinical & Academic	Public
P6	Female	Nurse	Researcher	Public
P7	Female	Emergency Physician	Joint staff: Clinical & Academic	Public
P8	Male	Physician	Researcher	Public
P9	Male	Emergency Physician	Academic	Public
P10	Female	Emergency Physician	Governance	Public
P11	Female	Public Health	Governance	Public
P12	Female	Nurse	Joint staff: Clinical & Academic	Public
P13	Female	Nurse	Joint staff: Clinical & researcher	Public
P14	Male	Emergency Physician	Researcher	Public
P15	Male	Emergency Physician	Joint staff: Clinical & Academic	Public
P16	Female	Nurse	Joint staff: Clinical & Academic	Private
P17	Female	Emergency Physician	Academic	Public
P18	Male	Emergency Physician	Joint staff: Clinical & Academic	Public
P19	Female	Emergency Physician	Researcher	Public
P20	Female	None	Primary health care site facilitator	Public
P21	Female	None	Community representative	Public
P22	Female	Nurse	Community representative	Public
P23	Female	Public Health Physician	Academic leadership	Public
P24	Female	Physician	Academic leadership	Public
P25	Female	Emergency Physician	Academic	Public
P26	Male	Physician	Postgraduate student	Public
P27	Female	Physician	Postgraduate student	Public
P28	Male	Physician	Postgraduate student	Public
P29	Female	Prehospital	Postgraduate student	Public
P30	Male	Prehospital	Postgraduate student	Public

Academic = Clinical Teaching

The two-stage sampling strategy resulted in a relatively large number of interviews. While data redundancy was reached for some topics earlier in the interview process, interviews were continued to capture the breadth and variability of perspectives across stakeholder groups and to ensure redundancy across all areas of interest. Ongoing data collection also allowed comparison and corroboration of perspectives across different stakeholder roles. Following the 30th interview, no new insights were identified across categories, and data redundancy was therefore considered to have been achieved. Given the richness of the dataset and the breadth of categories that emerged, findings were organised across separate focus areas. This paper focuses specifically on the composition, boundaries, and relationships within the WC EC community.

Analysis of the 30 interviews led to the development of three overarching categories: [1] Breadth, Variability, and Ambiguity within the Community; [2] Fragmentation and Exclusion; and [3] Engagement Potential and the Role of Frontline Clinicians.

Table 2 provides an extract of the coding tree, illustrating how meaning units were abstracted into categories. Participants were offered the opportunity to review their interview transcripts and the final results, however no additional feedback was received.

**Table 2:** Coding tree extract.

Meaning unit	Condensed meaning unit	Code	Sub-category	Category
“Anybody who deals with an emergency patient. So any specialists, orthopedic surgeons, anyone who's involved with the care or the management of the emergency patient?” – P16	Anybody who deals with an emergency patient... anyone who's involved with the care or the management.	The community is very broad	The community is broad	The community is broad and variably defined
“So it's almost as if the community has to has to be flexible for to what the intent is” – P10	So it's almost as if the community has to be flexible to the intent.	The community is flexible	Variably Defined Community	
“There probably wasn't a cohesive ongoing discussion between the academic community and the clinical community or the community within which the research was conducted in terms of the recipients of that research.” – P17	There probably wasn't a cohesive ongoing discussion between the academic community and the clinical community... or the community within which the research was conducted.	Limited community communication	Absent Communication	The community is fragmented
“The vehicles and the platforms that we have currently to engage with communities I think is ineffective and there's a lack of trust on both sides” – P15	The vehicles and platforms... to engage with communities... are ineffective and there's a lack of trust on both sides.	Lack of trust between community sectors	Lack of Trust	
“I feel like I have a moral imperative to be an engaged scholar, especially when there's limited resources” – P1	I feel a moral imperative to be an engaged scholar especially with limited resources.”	Awareness of need for engagement	Importance of engagement understood	Engagement is valued with frontline clinicians seen as key interfaces.
“I think between service platform and academia, as I said the service platform I think they're closer to the ground. They probably understand some priorities are, a bit better – P15	...the service platform... they're closer to the ground... understand some priorities... better.	Frontline workers know real-life priorities	Frontline Staff as a potential interface	

### Breadth, variability, and ambiguity within the community

#### The community is extremely broad

When asked who they believed made up the “community” in the WC EC setting, most participants emphasised its breadth. Some provided detailed lists of individuals and groups, while others gave broader descriptions, all reflecting the view that the EC community is extremely wide-ranging and spans diverse sectors and roles.

*“So I would say EMS, the non-clinical staff, the control room, the dispatchers, the disaster management, the academic supporting staff that are integrated and provide evidence. So it's incredibly broad” – P10*

*“So it can be very wide, and it should be very wide” – P4*

#### Variably defined community

Many participants felt the definition of the community was not fixed but varied with the research context. For some, it was shaped by the specific research question, while others emphasised geography as a central factor.

*“It becomes relevant in the research questions that we have to engage a different community in each case. Based on who the quote-unquote target of that research is” – P1*

*“when we're defining what the community is, that may change for a specific research question that we're asking” – P3*

*“We define the community as the folks living in that neighborhood” – P19*

A alternative perspective emphasised the importance of defining the community at the outset, before formulating research questions, to ensure that engagement is genuinely representative and not shaped solely by predetermined agendas.

*“But importantly, if we approach the community with a project, then we've already decided what questions are important. So I think it's a little bit of both in the sense if you're setting higher level agendas about important questions, then you need to think about being as representative as possible” – P1*

#### Unclear roles for some members

While certain stakeholders, such as emergency clinicians and prehospital providers, were consistently identified across interviews, the role of others appeared more contested. In particular, the role of patients emerged as a key point of divergence. Although most participants acknowledged that they had a role within the EC community, views differed on how central or consistent that role should be. Some believed patient input was essential and should be integrated into all stages of the research process.

*“I think most definitely that community members and patients should be involved in research. They are the people that are on the ground level, that are the sufferers so they will give you a clear and important idea of what the needs are…” – P22*

*“I think it's so important to understand what the people that we serve want or need or desire” – P11*

A commonly held view was that patient involvement in EC research was context-dependent, shaped by the specific aims and relevance of the research. Several noted uncertainties around how best to involve and define patients or what role they should play, particularly in studies less directly tied to patient experience. As such, decisions about patient engagement were seen as complex.

*“The one thing that I don't really have a solution for is patient involvement…. it's really hard to define who that patient is, because it could be anyone. It's not like a disease-specific condition where you're able to say these are the patients that I can pigeon hole a little bit. So I do think patients are part of that emergency care community, but I don't really know how to define those patients other than anyone.” – P2*

*“I think it should be a consideration rather than that they (patients) have to be included in every research project” – P7*

*“I think the patients could be excluded when it's going to be something that the patient involvement would have no influence on. ” – P20*

### Fragmentation and exclusion

#### Absent communication

Challenges with communication were raised consistently by participants, with many describing it as inadequate or ineffective. Stakeholders highlighted limited dialogue across sectors and expressed concern about the lack of sustained interaction between key groups.

*“There probably wasn't a cohesive ongoing discussion between the academic community and the clinical community or the community within which the research was conducted” – P17*

One of the most commonly cited reasons for these difficulties was the lack of regular in-person contact. Participants emphasised that physical distance between community members limited opportunities for discussion, relationship-building, and shared understanding.

*“I think the biggest problem is not really having a space or place to discuss the problems and help shape those into research questions because a lot of my experience has been that researchers use a very different language to frontline clinicians and managers and we don't always really gel.”*

*– P2*

*“So because we are kind of removed sitting at University X, far away from any hospital, we don't accidentally bump into practitioners, or we don't necessarily see what it looks like on the floor…. So I think its the physical, geographical distance between where some practitioners are and where some community members are.” – P6*

*“I don't think there's enough communication. They (researchers) don't come around and go like hey you that's working here, what do you need us to look at? What do you want to look at? What are the questions that you have?” – P4*

Additionally, some participants expressed concerns that alternative, indirect communication methods, such as email or phone calls, undermined trust, particularly in interactions with patients or the public, where face-to-face contact was viewed as essential for building credibility.

*“The problem is often we start our engagement with emails and then it takes forever to get a reply or people don't reply. And then you have to go back to them, or they send you an e-mail and then you don't reply…our engagements have been really delayed because every single time we need to move forward for another engagement, there's an issue with answering emails” – P1*

*“In person. In person. In person. That would be would be ideal. There isn't any other way for community….most of our communities are not on e-mail. They do not have a computer. They do not have a laptop.” – P21*

“*When you look at the track in government and trust with patients and family face to face always works because people don't trust the phone call*” – P11

#### Lack of trust

Alongside limited communication a broader lack of trust within the WC EC community was highlighted. This mistrust was reported across multiple levels, both internally within stakeholder groups and externally between different sectors. Participants attributed this to past experiences, competing agendas, and limited transparency, with some describing research as extractive or misaligned with community needs.

*“I'm not convinced that that relationship has been well established enough within all the domains within the community….if that relationship of trust doesn't exist, then it makes it quite difficult to align the different perspectives priorities and agendas” – P17*

*“I think academia and research, it's a backstabbing world and there's always this worry about what's their agenda, and why are they're doing this, and what do they want us to say…” – P8*

*“The vehicles and the platforms that we have currently to engage with communities I think is ineffective and there's a lack of trust on both sides, I think communities at every engagement that I've been to, they always will mention that theirs is a problem with trust, that we're coming to do research on them” – P15*

#### Absent voices

Several participants noted that certain members of the community, especially policymakers and those at the public level, were difficult to access. Without existing relationships or insider roles, engaging the right people was often a challenge. Safety concerns and structural barriers were also cited as limiting access to some communities.

*“If you don't come with pre-existing relationships or you’re in a post where you straddle service and research, it's really hard to get access to the right people….. it's hard to gain access to the policymakers if you do not have pre-existing relationships with them….Then it's even harder to get access to the right people when we talk about engaging with communities on the ground level” – P1*

*“ Safety to use one thing that just comes off the top of my head. If you needed to go into communities, to conduct research, number one is it safe to go into the area?” – P29*

Participants highlighted that many stakeholders outside academia faced competing clinical or operational priorities that limited their capacity to engage in research. This often created a false impression of disinterest, when time constraints and lack of meaningful inclusion were more likely barriers.

*“…the non academics don't really think of research too much because they're too busy with their general work” – P18*

*“Prioritisation of clinical responsibilities versus research is completely clear for most emergency clinicians as it should be. It's the patient in front of me, or there's the research project that might see daylight in two years time…” – P8*

*“I think the assumption is there's little interest, but I think that's probably a false assumption. It's just that if you're not invited to the party kind of thing” – P7*

As a result of these barriers, participants expressed concern that key community members were routinely excluded from research, despite their central roles in the EC system.

*“And even private GP's [non-specialist general practitioners from the private sector] I think are neglected and actually don't really sync very well with our system…..And then another profession would be the nurses….Then prehospital, and likewise different scopes of prehospital and public and private….And then probably a fourth one would be a broad term of administrators. I think we probably neglect and don't really involve in our research the guys who operationally, pull things together” – P8*

*“I think overall my sense would be they [patients] are too often ignored, which I think is the case for a lot of health systems work. You know, we tend not to incorporate patients perspectives or broader public perspectives into how we think about planning and doing research …” – P19*

#### Assumption of community need & misaligned priorities

The absence of certain community members from research discussions was seen to contribute to assumptions about their needs and priorities. This was felt to result in research agendas that were often misaligned with real-world experiences, with limited input from those directly affected informing project focus and design.

*“I think we often feel like we understand our community and so feel comfortable to speaking on their behalf and I think that may be a barrier to us actually stopping to really gain insights” – P14*

*“In actual fact we are only basing that off our own assumptions…. But at the end of the day that might actually not be what they [patients] want” – P30*

*“the guys working operationally are experiencing certain recurring problems and difficulties, but the people sitting at the university aren't necessarily doing research within those recurring kind of problems or issues” – P6*

### Engagement potential and the role of frontline clinicians

#### Growing recognition of engagement’s value

There was broad recognition of the value of engagement, with participants noting it had become more prominent in institutional narratives and departmental goals. This growing awareness was viewed as an encouraging foundation for future efforts.

*“I think engaged research is seen as a part of that [social responsiveness] and it's one of our targets or goals of the division of department and of the university….. I think research engaged scholarship is much more talked about now than five years ago. So I think the narrative is certainly going around. I think people realise that it's extremely important. It's a priority.” – P15*

*“We are the people that can actually do this and should be able to do this and could make time for this….” – P3*

*“If you try and pull clinicians in to do research, they get it, they see the need and they see the outputs…” – P8*

#### Frontline workers hold untaped knowledge

Frontline staff were described as holding valuable insights and awareness of available data sources. However, participants noted that this type of knowledge was often undervalued or overlooked in formal research processes.

*“sometimes the tacit knowledge within our healthcare systems from frontline providers or managers might not be considered academic but is an incredibly important type of knowledge that we really need…” – P19*

*“I think the clinical utility has been very clear to the clinicians for a long time but now we are starting to understand the richness of data that's there and how it can be really utilised” – P2*

*“…come, let's do some research. We've got so much information in our facilities and it just sits there”*– P16

Building on this, many interviewees felt that frontline staff’s direct exposure to system challenges gave them a strong understanding of research priorities.

*“ …Whether it's middle management, you look at the academics as the ones who spew out the knowledge and the wisdom.But actually the people on the floor or in the ambulance or wherever they are, they are experiencing it first-hand. …we could get the actual information from the people who are actually living it”. – P16*

*“While the clinicians very much are aware of what needs to be researched or what questions need to be answered, they're not the ones who are driving the actual research activities” – P17*

#### Frontline clinicians bridging the gap

Frontline clinicians, especially those working in joint academic and clinical roles, were identified as very well placed to act as bridges between overlooked community members and the academic sphere. Their dual positioning was seen to offer a clearer understanding of service-level priorities and on-the-ground realities, allowing them to help align research agendas with the needs of the broader community. Participants described these roles as uniquely suited to fostering cross-sector engagement and reducing barriers to meaningful scholarship.

*“I think the closer to the community, for example the people working on ground level, working in primary health care, in emergency medicine, in the centers, the closer you work to the community, the more closer to the truth your list of priorities will be” – P15*

*“But I think the joint staff are actually in a particularly sweet spot because they have one foot in the in academia and one foot within service…They are right there in the forefront and can actually be part of both understanding, what the key research questions are and actually part of answering those research questions…….Joint staff in particular are a really unique mechanism for us to shortcut some of the barriers that are usually associated with engaged scholarship, particularly in terms of engagement with other sectors, because in this instance you're wearing two hats. So you are an insider on both sides of of the coin, so to speak” – J1*

## Discussion

Defining the EC community was complex, with stakeholders emphasising its breadth and variability aligning with the wider sociological literature that describes “community” as a fluid, contested and multidimensional concept, rather than a fixed entity [32,33]. This challenge has been reported in studies examining themes and needs within community-engaged research previous, where communities are described as heterogeneous and cannot be assumed to speak with a single voice [34]. The prevailing view on engaged scholarship, and related approaches, is that communities should be identified before research questions are formulated, ensuring inclusivity and relevance [[34], [35], [36], [37]]. This perspective was reflected by many of our participants, who emphasised the need to define the community at the outset. However, some argued that beginning with the research question may help to identify which stakeholders should be involved and to what degree, particularly when boundaries are otherwise unclear. Although this challenges the dominant model, it is consistent with the understanding of engaged research as an iterative process [36]. In this view, defining the question first may be acceptable in certain circumstances, such as those described by Wallerstein et al. [35], where research questions build on prior collaborative projects, and where researchers may approach communities to gauge interest in co-developing solutions to known problems.

Our findings that certain stakeholder voices were perceived as absent or unclear within research processes is supported by the academic literature. A mismatch between patient priorities and researcher focus has previously been demonstrated [38] and a recent systematic review of 150 consecutive randomised clinical trials in high-impact medical and surgical journals found that only 5% reported patient or family representation in their design or conduct [39]. The engagement with other key groups, including frontline clinicians and decision makers, appears to be even lower. One review cataloguing methods of stakeholder engagement reported that while 80 % of included studies described engagement with patients and the public, fewer than half (45.7 %) reported involving healthcare providers, and under a quarter (22.8 %) included policy makers [40]. Beyond the absence of certain groups, our findings also reflected uncertainty around the roles these stakeholders should play and how best to integrate them into research. Reviews of stakeholder engagement highlight diverse approaches with no agreed method and consistently report limited detail on the processes used [[41], [42], [43]]. Where engagement methodologies are reported, clear shortcomings are often identified. One review of patient and public involvement in health research in LMICs found that a significant number of studies were limited to “consultation” (decision-making informed by participants’ views), rather than deeper forms of on-going collaboration or user-controlled research [44]. Additional concerns raised in the literature include stakeholder involvement being restricted to isolated stages rather than sustained across the entire research process [41], and an over-reliance on community “gate-keepers” instead of closer engagement with end users [44]. Our findings align with previous research, underlining the need for clearer guidance on the processes of engaged scholarship, including how and when to involve stakeholders, as well as the structures that can support this engagement.

Participants in this study described the WC EC community as fragmented and marked by limited trust, a challenge also noted in other investigations of barriers to engagement. Mistrust has been shown to arise from communities’ prior experiences with research and healthcare, with the formation of effective partnerships shaped by the extent to which communities have historically felt marginalised [34]. This is particularly salient in the South African context, where this mistrust cannot be separated from historical legacies, including colonialism and apartheid, which have contributed to enduring inequities in healthcare access, entrenched hierarchies, and perceptions of research and academic institutions as distant from community partnership for some groups. Hierarchical relationships between healthcare professionals and patients specifically have been identified as a barrier to meaningful participation, in contrast to the facilitative role of relationships that are equal, respectful and informal [45]. Trust itself has been repeatedly highlighted as essential for successful engagement in reviews of facilitators of community-engaged research [46,47] and building partnerships may therefore require “relationship repair” to address the harms and mistrust associated with past research encounters [34]. Such repair extends beyond individual interactions and requires sustained attention to power, reciprocity, and meaningful inclusion within engagement processes. Our findings add weight to calls for further work to understand how trust and social capital can be measured and actively fostered within community–academic partnerships, as advocated in previous studies [46,48].

Frontline clinicians were consistently highlighted in our study as potentially key, underutilised stakeholders in engaged scholarship. Their involvement has been recognised in the literature, noting that frontline staff can convey the everyday decision-making challenges they face in practice, providing a basis for new and relevant research questions. They also bring extensive clinical expertise and first-hand knowledge of delivering care, which represents the kind of real-world insight that research ultimately seeks to inform [49]. More active involvement of a broad range of clinical stakeholders during the planning stages has been suggested as a way to avoid common barriers to trial implementation and to improve the translation of findings into practice [50]. Despite these advantages, frontline clinicians are still infrequently included in engaged scholarship initiatives. Many report perceiving research as outside their professional remit and describe feeling detached from ongoing academic activity [51]. Several studies have drawn attention to the obstacles that limit clinician involvement in research, including competing clinical demands, lack of protected time, and uncertainty about their role within research partnerships [49,51,52]. When dedicated projects have made a specific effort to involve clinicians, successful collaboration has been demonstrated; however, the resulting interventions were described as not sustained over time [53]. This points to the need for further work on how best to embed and support clinician participation within engaged scholarship. Frontline clinicians hold particular promise as they are uniquely placed to link the realities of everyday practice with academic research, helping to ensure that study questions and outcomes remain aligned with service priorities and patient needs. Building on this potential, our findings suggest that frontline clinicians can play a pivotal role in strengthening engaged research and, importantly, in serving as a bridge between academic teams and the wider community.

As with all qualitative studies, this work is shaped by participants’ perspectives and the researchers’ positionality. SSIs reflect the accounts of those who agreed to participate, raising the possibility of bias, particularly as individuals already engaged with research may have been more likely to take part. The research team were active members of the WC EC community, which may have influenced both participants’ responses as well as interpretations of findings. Reflexive practices, including maintaining records and team debriefings, were used to critically examine assumptions and minimise bias [54].

## Conclusion

Our study highlights the complexity of defining the EC community, with boundaries and membership often broad and contested. Key stakeholders, including patients, decision-makers and frontline clinicians, remain absent or their roles unclear in many research processes. Trust was identified as central to meaningful engagement, yet fragmentation continues to undermine partnerships. Frontline clinicians were viewed as underutilised, despite their ability to link everyday practice with academic inquiry. Supporting their involvement, alongside clearer guidance on engagement processes, is essential to strengthen engaged scholarship and enhance research relevance.

## Dissemination of results

Results from this study have been shared with all participating stakeholders via email. Preliminary findings have already been presented at regional and international conferences. The aim is to present the full results of this study, alongside findings from related research, at upcoming WC strategic meetings and forums at both regional and international levels.

## CRediT author statement

*RH*: Conceptualisation, Methodology, Validation, Formal analysis, Investigation, Data Curation, Writing- Original draft preparation, Visualisation, Project administration. *WS*: Conceptualisation, Methodology, Validation, Writing - Review & Editing, Supervision *CS:* Conceptualisation, Methodology, Validation, Investigation, Writing - Review & Editing, Supervision.

## Declaration of competing interest

Authors RH and WS hold editorial roles with the African Journal of Emergency Medicine. Neither were involved in the editorial workflow for this manuscript, and all journal peer reviews are conducted through a double-blinded process. The authors declare no further competing interests.
